# Multi-target QSAR modelling in the analysis and design of HIV-HCV co-inhibitors: an in-silico study

**DOI:** 10.1186/1471-2105-12-294

**Published:** 2011-07-20

**Authors:** Qi Liu, Han Zhou, Lin Liu, Xi Chen, Ruixin Zhu, Zhiwei Cao

**Affiliations:** 1 College of Life Science and Biotechnology, Tongji University, 200092, China; 2 School of Computer Science, Carnegie Mellon University Pittsburgh, USA

## Abstract

**Background:**

HIV and HCV infections have become the leading global public-health threats. Even more remarkable, HIV-HCV co-infection is rapidly emerging as a major cause of morbidity and mortality throughout the world, due to the common rapid mutation characteristics of the two viruses as well as their similar complex influence to immunology system. Although considerable progresses have been made on the study of the infection of HIV and HCV respectively, few researches have been conducted on the investigation of the molecular mechanism of their co-infection and designing of the multi-target co-inhibitors for the two viruses simultaneously.

**Results:**

In our study, a multi-target Quantitative Structure-Activity Relationship (QSAR) study of the inhibitors for HIV-HCV co-infection were addressed with an in-silico machine learning technique, i.e. multi-task learning, to help to guide the co-inhibitor design. Firstly, an integrated dataset with 3 HIV inhibitor subsets targeted on protease, integrase and reverse transcriptase respectively, together with another 6 subsets of 2 HCV inhibitors targeted on NS3 serine protease and NS5B polymerase respectively were compiled. Secondly, an efficient multi-target QSAR modelling of HIV-HCV co-inhibitors was performed by applying an accelerated gradient method based multi-task learning on the whole 9 datasets. Furthermore, by solving the *L*-1-infinity regularized optimization, the Drug-like index features for compound description were ranked according to their joint importance in multi-target QSAR modelling of HIV and HCV. Finally, a drug structure-activity simulation for investigating the relationships between compound structures and binding affinities was presented based on our multiple target analysis, which is then providing several novel clues for the design of multi-target HIV-HCV co-inhibitors with increasing likelihood of successful therapies on HIV, HCV and HIV-HCV co-infection.

**Conclusions:**

The framework presented in our study provided an efficient way to identify and design inhibitors that simultaneously and selectively bind to multiple targets from multiple viruses with high affinity, and will definitely shed new lights on the future work of inhibitor synthesis for multi-target HIV, HCV, and HIV-HCV co-infection treatments.

## Background

Human immunodeficiency virus (HIV-1) is the cause of acquired immunodeficiency syndrome (AIDS) which has infected more than 60 million people around the world [1,2]. Meanwhile, Hepatitis C virus (HCV), which is served as a serious cause of chronic liver disease, has infected 150-200 million people worldwide [3]. Nowadays HIV and HCV infections have become global public-health threats. Even more remarkable, HIV-HCV co-infection is rapidly emerging as a major cause of morbidity and mortality throughout the world, since that both of the viruses share the same routes of transmission [3,4]. It is shown that infection with the HCV is the most common co-infection in people with HIV, and hepatitis C is categorized as an HIV-related opportunistic illness. Complications related to HIV-HCV co-infection are becoming an increasingly important medical issue [4].

The current strategies for developing HIV/HCV antiviral agents depend essentially on disrupting the replication of the 2 viruses, and various inhibitors have been designed to target and block the functions of the enzymes necessary in the replication cycle of HIV/HCV. Among them, HIV inhibitors commonly target on protease, integrase and reverse transcriptase (RT), while HCV inhibitors target on NS5B polymerase and NS3 serine protease [5-18]. These inhibitors have been considered as attractive targets for therapeutic intervention in HIV/HCV infected patients.

For HIV and HCV therapy, single antiretroviral drug, alone or in simply combination with each other, is no longer recommended for clinical use owing to (1) the complicated infection mechanism of these two viruses; (2) the severe side effects of the joint using and (3) the rapid emergence of drug-resistant strains after initiation of therapy. Hence, drugs targeting on different targets with high therapeutic and reduced side effects are expected to be more effective at suppressing viral growth. For HIV, The multi-target antiretroviral drugs can succeed in inhibiting several HIV proteins simultaneously and efficiently. There has existed several pioneering work in multi-target drug discovery for HIV infection, such as the multi-target antiretroviral drug Cosalane [13], which was developed to inhibit several HIV-1 proteins simultaneously. Compared to HIV, the multiple target HCV drug treatment is still in its infancy. Nevertheless, the combination use of single-target HCV drugs has become a new chance in this field, such as the combination using of NS5B polymerase inhibitor (GS-9190) and NS3 protease inhibitor (GS-9256), which were shown to be safe, well-tolerated and show dose dependant antiviral activity [19,20].

Since for both HIV and HCV the small-molecule compounds used to design the drugs are needed to be assayed in vitro and in vivo, the popular in-silico Quantitative Structure-Activity Relationship (QSAR) modelling is applied extensively in HIV/HCV inhibitor studies due to its charming "black-box" characteristics as well as its well prediction ability. Normally the QSAR modelling can be viewed as a computational technique to elucidate a quantitative correlation between chemical structure and biological activity [21]. Recently, considerable QSAR studies have been made for HIV/HCV inhibitors studies [5-18]. However, these studies were mainly focused on specific types of targets or specific diseases individually. Few studies have been performed on the multi-target HIV-HCV co-infection QSAR modelling. Although the ways in which co-infection with HIV and HCV affect the body are still poorly understood, it has been indicated that both HIV-1 Protease and HCV NS3 Protease are responsible for cleaving the viral polyproteins during the course of their action to produce the individual proteins of the mature viruses. Similarly, HIV-1 reverse transcriptase and HCV NS5B can be affected by either nucleoside inhibitor that terminates nucleic acid synthesis or non-nucleoside inhibitor that impairs enzymatic function [22,23]. All these evidences have indicated that it is possible to design certain inhibitors that aim at both HIV targets and HCV targets simultaneously. From this point of view, multi-target co-infection QSAR modelling for HIV and HCV is attractive and promising, due to that it is easy to achieve and expected to provide useful clues on how to synthesize such co-inhibitors with improved affinities.

In our previous study, we presented a multi-target QSAR modelling on HIV-1 inhibitors individually [31]. In this study we desire to extend this model to investigate the multi-target QSAR modelling of HIV and HCV jointly and simultaneously, and aim at providing useful clues on the design of HIV-HCV co-inhibitors. The QSAR modelling of HIV-HCV co-infection inhibitors (co-inhibitors for short) was addressed by applying an efficient accelerated gradient method based multi-task learning (MTL) model provided by us formerly in machine learning community [24]. QSAR studies were performed on 9 datasets of HIV and HCV inhibitors. By using our MTL framework, the correlations among different set of inhibitors were utilized and an efficient multi-target QSAR modelling of HIV-HCV co-inhibitors was obtained. According to the importance of each descriptor in QSAR model, the Drug-like index (DL) features [25] for inhibitor description were ranked, and a drug structure-activity simulation were performed to investigate the relationships between compound structures and binding affinities based on the ranked molecule descriptors.

## Methods

### A Dataset

Our integrated dataset contains 3 kinds of HIV target subsets and 6 kinds of HCV target subsets, which were compiled from a thoroughly literature reviewing, consisting of inhibitors with their binding affinities on HIV protease, integrase and reverse RT, as well as HCV NS3 and NS5B respectively. This data provided the first time a comprehensive data source for multi-target HIV-HCV co-infection QSAR study. In our study, these inhibitors are correspondingly referred as (1) protease inhibitors, which prevent HIV from processing and packaging new virulent viral particles, (2) integrase inhibitors, which inhibit the proviral DNA to insert into the host cell genome, (3) non-nucleoside reverse transcriptase inhibitors (NNRTI), which inhibit the virus by preventing the transcribing of its genomic DNA into proviral DNA for incorporation into the host cell DNA, (4) NS3 serine protease inhibitors, which prevent polyprotein processing and restore the hepatocytes innate antiviral response, and (5) NS5B polymerase inhibitors, which prohibit the synthesis of RNA strands of HCV. All the enzymes affected by these inhibitors have been reported as the most important targets for chemotherapeutic agents against the diseases caused by HIV/HCV. General descriptions of inhibitors for these targets were listed in Table 1.

**Table 1:** Dataset descriptions.

Dataset ID	Target type	Number of inhibitors	Activity measurement
1	HIV-1 Reverse Transcriptase	79	EC_50 _[37]

2	HIV-1 Integrase	213	IC_50 _[6]

3	HIV-1 Protease	106	pKi [1]

4	HCV NS5B Polymerase	67	IC_50 _[7]

5	HCV NS5B Polymerase	45	IC_50 _[8]

6	HCV NS5B Polymerase	41	EC_50 _[9]

7	HCV NS3 Serine Protease	42	pKi [10]

8	HCV NS3 Serine Protease	53	pKi [9]

9	HCV NS3 Serine Protease	34	EC_50 _[11]

Similar to our previous study [31], the inhibitors were represented with 2 kinds of feature spaces referring to 32-dimensional General Descriptor (GD) features and 28-dimensional Drug-like index (DL) features. Although there are numerous types of descriptors to describe a chemical compound, none of a set of descriptors can guarantee to behave overwhelming better than others. Therefore, the widely applicable set of descriptors, *i.e*., the GD [25] was selected, together with the DL descriptor [26,27] as a complement.

Detailed biological meaning of GD and DL descriptors can be referred in our previous work [31]. It should be noted that: (1) normally, general descriptors characterize physical prosperities of compounds, while drug-like index descriptors characterize simple topological indices of compounds. These two kinds of descriptors are expected to present a comprehensive description of the compounds from the views of their intrinsic characteristics as well as their drug-like properties. (2) The GD descriptor is generated in a hybridized way thus its current features haven't kept their original means for compound structure description. Therefore it cannot be biologically explained easily. On the other side, DL holds its original meanings, thus will be applied in our following feature ranking and explanations.

It was shown in Table 1 that the inhibitor activity of the molecules were measured with *EC_50_*, *IC_50 _*or *PK_i_*, which are the most commonly used measurements of the compound inhibitions [28]. *EC_50 _*(half maximal effective concentration) refers to the concentration of a drug, antibody or toxicant which induces a response halfway between the baseline and maximum after some specified exposure time, while *IC_50 _*(half maximal inhibitory concentration) refers to the concentration it needed to inhibit a given biological process or component of a process by half. *IC_50 _*can be converted to the *PK_i _*measurement by the Cheng-Prusoff equation:(1)

Where [*L*] is the concentration of free radio ligand used and *K_D _*is its equilibrium dissociation constant for the receptor [29].

It should be noted that the QSAR data were provided by different research groups under different platforms/protocols with different activity measurements. Normally QSAR modeling achieved by such single target data is often not reliable due to the insufficiency of samples. However, since we want to investigate the multi-target QSAR relationship of the HIV-HCV co-infection, these data can be integrated in an elegant multi-target QSAR model taking the advantages of the multi-task learning [30], which would expect to exploit the possible synergies between different datasets and obtain a better QSAR model to guide the synthesis of certain inhibitors with enhanced activities for HIV and HCV simultaneously. Details will be shown in the following.

### B Methodology

#### Computational framework for multi-target modelling

**Figure 1:**
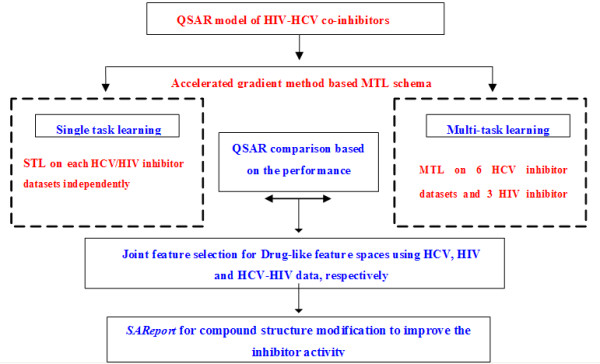
**The general computational framework for multi-target QSAR modelling of HIV-HCV co-inhibitors**.

The general computational pipeline for our study was presented in Figure 1. In our previous study we have presented the first time a multi-task learning algorithm for cross-platform siRNA efficacy prediction [30], and also utilized such MTL-based model for feature-selection in HIV-1 QSAR modeling [31]. It has been proven to be more effective than learning each QSAR modeling on single target independently [31]. Some latent commonalities across tasks can be exploited through MTL, which is expected to boost the learning performance of each single task.

In current study, a novel accelerated gradient descent algorithm based MTL model was performed for multi-target QSAR modeling on our integrated datasets simultaneously. Our in-house experiments indicated that this MTL model is more efficient than our formerly adopted one for multi-target QSAR modeling [31] and it is scaled up well for large scale QSAR modeling in both convergence speed and learning accuracy. A joint *L*-1-infinity regularization based feature selection procedure was performed on the DL feature space to reveal the most common features across multi-target HIV-HCV co-infection QSAR modeling. Based on such model, a drug structure-activity simulation for investigating the relationships between compound structures and binding affinities was further presented to validate our selected important features for efficient co-inhibitor synthesis and design.

#### Multi-task learning for QSAR modelling of HIV-HCV co-inhibitors

Multi-task learning has been developed in machine learning research to situations where multiple related learning tasks are accomplished together. It has been proven to be more effective than learning each task independently when there are explicit or hidden interrelationship among the tasks that can be exploited. The intuition underlying the framework is that the multiple related tasks can benefit each other by sharing the data and features across the tasks, which can often boost the learning performance of each single task [30]. Also it provides an efficient mechanism for cross-task feature selection, thus could uncover the common dominate features for all the tasks simultaneously. Such computational ability is inherently suitable for our multi-target QSAR modeling, in which each single QSAR model could be viewed as a task and the leading features for synthesizing co-inhibitors with improved activity will be identified under such schema.

It should be noted that the QSAR modelling is the process by which chemical structure is quantitatively correlated with a well-defined process, such as biological activity or chemical reactivity. And this procedure is generally formulated as a regression model [32] to predict the compound activity based on a given set of molecule descriptors. Although various statistical and machine learning methods have been proposed in the last few years for QSAR modeling [32], few studies have been tried in the multi-target QSAR scenario. In our study the multi-target QSAR modeling will be elegantly formulated as a multi-task regression framework to reveal useful clues for multi-target drug screening and synthesizing for HIV-HCV co-infections.

Basically in our multi-target modeling, assuming the datasets contains *N *tuples, z*_i _*= (x*_i_*, *y_i_*, *k_i_*) for i = {1...*N*}, where x*_i _*∈ R*^d ^*is the inhibitor descriptor and *k_i _*∈ {1...*M*} is the indicator specifying to which of the *M *task the example (x*_i_*, *y_i_*) corresponds to. A critical issue in this case is to learn a set of sparse functions across these tasks for activity regression. Specifically, the features will be represented as GD or DL. *y_i _*is the corresponding inhibitor binding affinity. Our goal is to predict the target binding affinity from a set of compound with known affinity by learning *M *linear regressions of the form . In our study the following square loss function is adopted:(2)

where *z *= (x, *y*, *k*), *W *=[w_1_, w_2_, ..., w*_M_*] ∈ R*^d × M ^*and *W^j ^*be the *j*th row of *W*.

In sparse MTL for features selection, we enforce the joint sparsity across different tasks by adding the *l*_1,∞ _norm of the matrix *W *to the square loss function, which leads to only a few non-zero rows of *W*, and thus the corresponding features will be used for prediction. In short, the optimization target function is defined as *F*(*W*), and we want to minimized the following function as:(3)

where(4)

and(5)

The *l*_1,∞ _norm of the matrix *W *is defined as:(6)

The first term in Equation (3) is the average of the empirical error across the tasks. The second term is the *L*-1-infinity regularization term that works on feature selection task in MTL, which can yield joint sparsity on both the feature level and task level and can lead to a more sparse solution [24].

As the main difficulty for solving the *l*_1,∞ _regularized formulation in formation (6) lies in the non-smooth property of the *l*_1,∞ _regularizer, we present an accelerated gradient descent algorithm with the convergence rate O (1/*t*^2^) by a variation of Nesterov's method calling a black-box oracle in the projection step at each iteration [24]. By exploiting the structure of the *l*_1,∞ _ball, we find the black-box oracle can be efficiently solved by a simple sorting procedure. Compared with Nesterov's algorithm, our method is suitable for large-scale multi-task learning problem since it only utilizes the first order information and is very easy to implement. Experiment results in our previous study have shown that our method significantly outperforms the most state-of-the-art methods in both convergence speed and learning accuracy [25].

Details of the accelerated gradient descent algorithm were presented in Algorithm 1. The generalized gradient update step was defined as following:(7)

where ||·||*_F _*denotes the Frobenius norm and 〈*A*, *B*〉 = Tr(*A^T ^B*) denotes the matrix inner product.

**Algorithm 1: **Accelerated Gradient Algorithm

Initialization: *L*_0 _> 0, *η *> 1, *W*_0 _∈ R^*d *× *M*^, *V*_0 _= *W*_0 _and *a*_0 _= 1.

Iterate for *t *= 0, 1, 2,... until convergence of *W*_t_:

1) Set *L *= *L*_t_

2) While *F*(*q_L_*(*V_t_*)) >*Q_L_*(*q_L_*(*V_t_*), *V_t_*)

*L *= *η L*

3) Set *L*_*t*+1 _= *L *and compute

In addition, we suggest a look-ahead stopping criterion for Algorithm 1 by firstly fixing a step size *h *and in each iteration *t *calculating the following ratio:(8)

We stop the procedure when *κ *≤ *τ *where *τ *is a prefixed constant.

Then, we discuss how to solve the generalized gradient update efficiently. Rewrite formulation (7), we obtain that:(9)

For the sake of simplicity, we denote  as *V *and  as . For equation (9) the following form was taken:(10)

where *W^i^*, *V*^*i *^denotes the *i *th row of the matrix *W*, *V *respectively. Therefore, (10) can be decomposed into *d *separate subproblems of dimension *M*.

For each subproblem:(11)

Since the conjugate of a quadratic function is still a quadratic function and the conjugate of the norm is the barrier function, the dual of (11) takes the following form:(12)

and the vector of dual variables *α *satisfies the relation *α *= v - w. Equation (12) can be solved by an efficient projection onto the ball *l*_1 _according to [33]. With the primal dual relationship, we present Algorithm 2 for solving (11):

**Algorithm 2: **Algorithm for projection onto the *l*_∞ _ball

**Input: **A vector v ∈ R*^M ^*and a scalar 

1) If ||v||_1 _, set w = 0. Return.

2) Let *u_i _*be the absolute value of *v_i_*, i.e. *u_i _*= |*v_i_*|. Sort vector u in the decreasing order: *u*_1 _≥ *u*_2 _≥ ... ≥ *u_M_*

3) Find 

**Output: **

#### Feature selection across multiple tasks

It has been demonstrated that the coefficient among different tasks will achieve zeros simultaneously by using the *l*_1,∞ _norm regularization with the "grouping" effect [24]. So the *l*_1,∞ _regularization can provide an efficient way to evaluate the joint feature importance in HIV-HCV co-inhibitor design across multiple target enzymes. The basic idea behind this joint feature selection procedure is that the parameter *W *outputted from the algorithm for projection onto the *l*_1,∞ _ball would present a ranking of the features based on their joint importance across multiple tasks. Formally, based on the parameter *W *derived from Algorithm 2, we can obtain *β_i _*according to the following equation(13)

The value of *β_i _*indicates the weight of the corresponding feature, which gives us a quantitative way to evaluate the importance of various features for HIV-HCV co-inhibitor design and synthesize.

#### Domain of applicability of the model

The domain of application (DOA) is an important issue for QSAR model which is used for estimating the reliability in the prediction of a new compound [34]. Those molecules which fall out the domain may lead to unreliable predictions. Extent of extrapolation is a simple method to define the DOA[35]. It is based on the value of leverage *h_i _*define in equation (14) for each chemical molecule:(14)

Where *X_i _*is the row-vector descriptor of the query compound, *X_i _*is the *n *× *k *matrix containing *k *descriptor values and *n *training samples. The superscript ***T ***refers to the transpose of the matrix or vector. Generally, the warning leverage h* is fixed at 3 *k*/*n*, where *n *is the number of training compounds, and *k *is the number of descriptor. When a leverage is greater than the warning leverage h*, the predicted activity is the result of substantial extrapolation of the model and, therefore, it may not be reliable.

Based on the definition of leverage, Williams plot was used in our study to visualize the DOA of the QSAR model [35]. The Williams plot plots the standardized cross-validated residuals (RES) versus leverage values (*h*), and can be used to obtain an immediate and simple graphical detection of both the response outliers (*Y *outliers) and the structurally influential chemicals (*X *outliers) of a model. Generally, the points with their values of *Y *axis fall outside the 3*σ *line (*σ *is the standard residuals unit of the compounds) can be referred as the *Y *outliers, while the points with their values of *X *axis fall outside the warning leverage h* line can be referred as the *X *outliers.

#### Multi-target HIV-HCV co-inhibitor design based on drug structure-activity prediction

After the feature ranking together with the examination of domain of application for multiple HIV-HCV drug targets QSAR modelling, a drug structure-activity prediction [27] was performed for the analysis of the multiple drug data. The goal of this study is two folds: (1) It is used to computationally validate the ranking result by our multi-task feature selection, and (2) It provides several useful modification strategies for further HIV-HCV co-inhibitor design.

**Figure 2:**
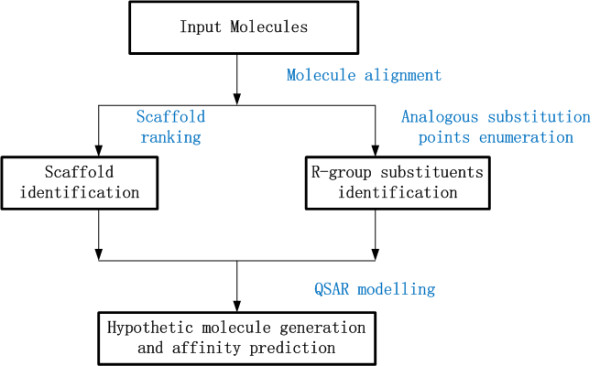
**Computational pipeline for structure-activity simulation and hypothetical molecule generation**.

**Figure 3:**
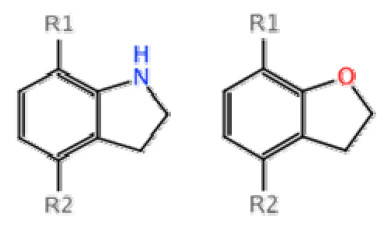
**An example of analogous substitution points**. The positions indicated by R1 for each molecule are considered analogous substitution points as the positions indicated by R2.

Our prediction pipeline is carefully designed as shown in Figure 2. To be brief, the whole procedure is achieved by the following steps: First, All the compounds used for our multi-target QSAR study are gathered as the input, to generate their common scaffolds. If more than one scaffold is presented, they are topologically aligned to produce a common numbering system. Second, assigning the scaffolds by enumerating all possible substructure matches, then minimizing a pair-wise energy term which leads to the lowest possible diversity of implied *R*-group substituents, which is expected to provide a set of analogous substitution points. An example in this case (Figure 3), the positions indicated by *R*1 for each molecule are, for this example, considered analogous substitution points, as are the positions indicated by *R*2. These positions have common meaning for the molecules, regardless of which of these two scaffolds they are based on. Finally, the list of hypothetical molecules, constructed from available ones, is generated by enumerating all of the input molecules, and performing single-point mutations at each of the substituent positions, with each of the *R*-groups that have been observed in the analogous position for some other molecule in the input dataset. The unique list of chimeric molecules is then scored according to an estimate of probability, scaled and balanced to match the distribution of activities found in the input set. The scores are scaled such that a value of 0 indicates that the hypothetical molecule is as likely to be active as an average molecule in the input set, while positive values are more likely [27].

Based on this computational prediction pipeline, we will identify what is the most efficient compound modification strategy to improve the molecule affinity targeting on multiple HIV-HCV enzymes. Also, these identified strategies will be further explained by our joint feature ranking obtained under the multi-target QSAR paradigm.

## Results and Discussions

Formulated as a multi-task regression problem, the QSAR modelling of HIV-HCV co-inhibitors was performed based on the accelerated gradient descent based sparse multi-task learning. Root mean squared error (RMSE) and squared correlation coefficient (*R*^2^) were adopted as the performance evaluation for testing results. The definitions of these statistical parameters are provided as followed:

Root mean squared error (RMSE):(15)

where *n *is the number of predicted drug molecules

, is the difference between the observed molecule affinity data and the fitted model

*y_i _*is the observed molecule affinity

 is the predicted molecule affinity

Squared correlation coefficient (*R*^2^):(16)

where *P^avg ^*is the average value of over the *n *predicted molecule affinities.

**Figure 4:**
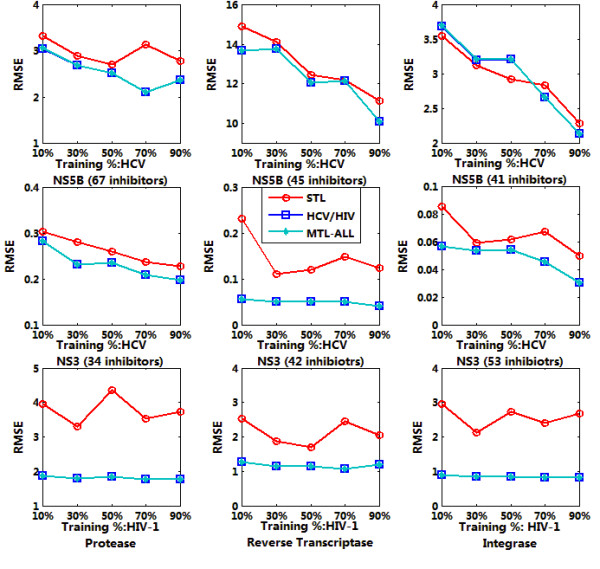
**RMSE comparison of QSAR modelling based on accelerated gradient descent based MTL with STL on 9 targets, described in GD representation**.

**Figure 5:**
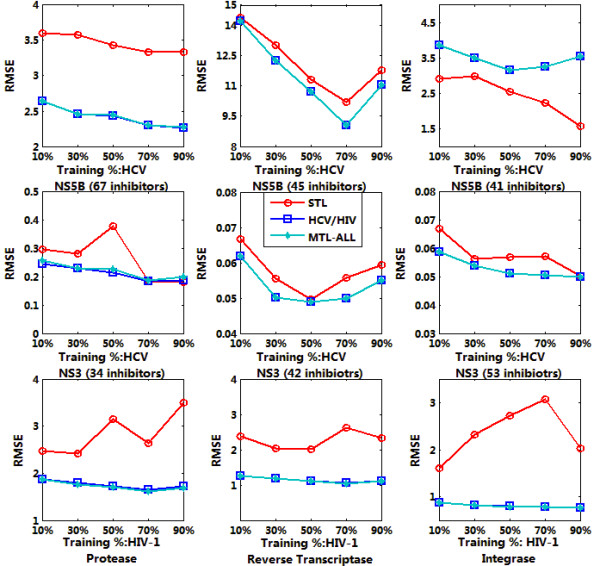
**RMSE comparison of QSAR modelling based on accelerated gradient descent based MTL with STL on 9 targets, described in DL representation**.

**Figure 6:**
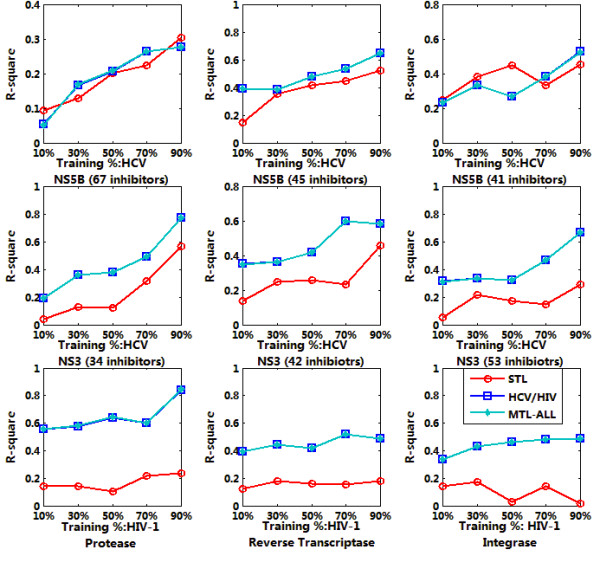
**R^2 ^comparison of QSAR modelling based on accelerated gradient descent based MTL with STL on 9 targets, described in GD representation**.

**Figure 7:**
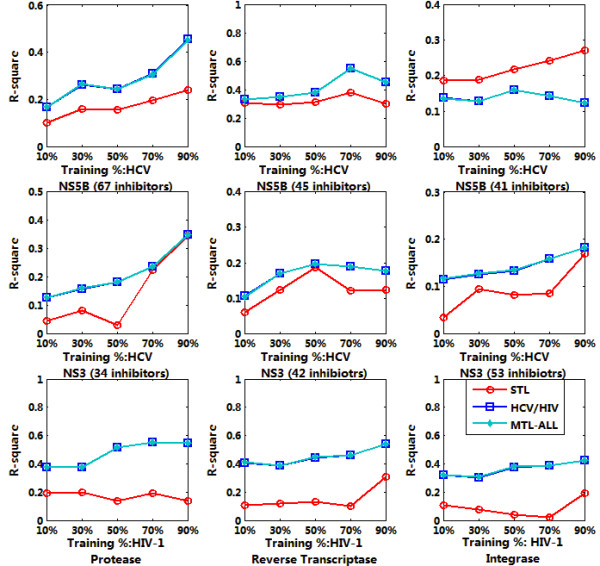
**R^2 ^comparison of QSAR modelling based on accelerated gradient descent based MTL with STL on 9 targets, described in DL representation**.

Sparse MTL was trained jointly on 9 targets datasets as indicated in Table 1 with 10%, 30%, 50%, 70% and 90% of the whole data respectively. The trained model was then applied for the affinity prediction of remaining data. We run each experiment 10 times and output the average RMSE and *R*^2^. For each target, the output were calculated based on single task learning (denoted as STL in the figure), multiple task learning on HCV or HIV (denoted as HCV/HIV in the figure) and multiple task learning on all the inhibitors (denoted as MTL-ALL in the figure). The testing results were summarized in Figure 4, 5, 6 and 7, denoting the scenarios of representation of compounds with GD and DL respectively. The goal of this test was to compare the performance of MTL and single-task learning (STL) in the QSAR modelling of HIV/HCV inhibitors.

It is obvious that for both GD and DI feature space, using multi-task learning for QSAR modelling is superior to single-task learning on most target datasets, with the evaluation of RMSE and *R*^2 ^and significant statistical confidence (data not shown). And the average correlation coefficient for data prediction under MTL is up to 0.6~0.7, which is a well-accepted QSAR results. Such results proved that multi-task learning can discover the latent commonalities across different types of inhibitors and take advantage of the synergy among multiple tasks when the label data on each single task are insufficient. These results also indicated that multi-task learning provides an effective way to boost the learning performance of each single task by exploiting the available synergy between them, thus served as an efficient paradigm for multi-target QSAR modelling.

**Figure 8:**
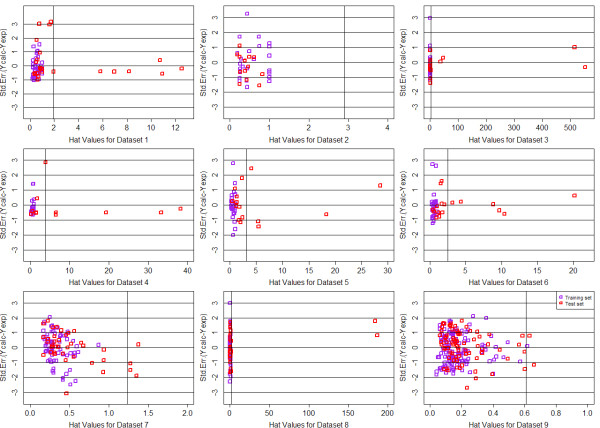
**Williams plots of 9 QSAR models based on MTL with Drug-like Descriptor**.

**Figure 9:**
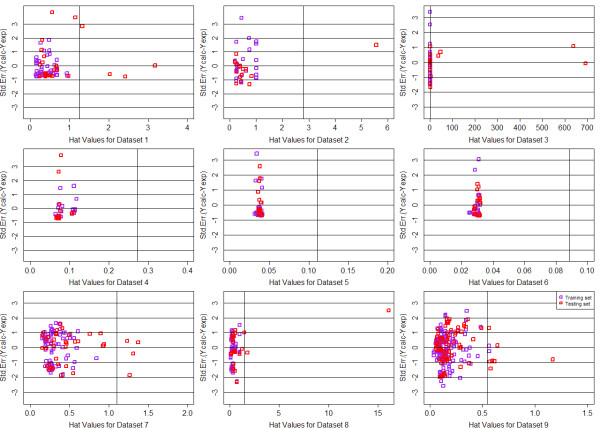
**Williams plots of 9 QSAR models based on STL with Drug-like Descriptor**.

**Figure 10:**
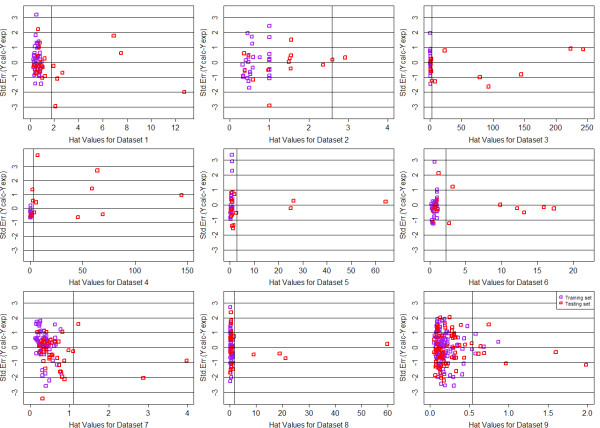
**Williams plots of 9 QSAR models based on MTL with General Descriptor**.

**Figure 11:**
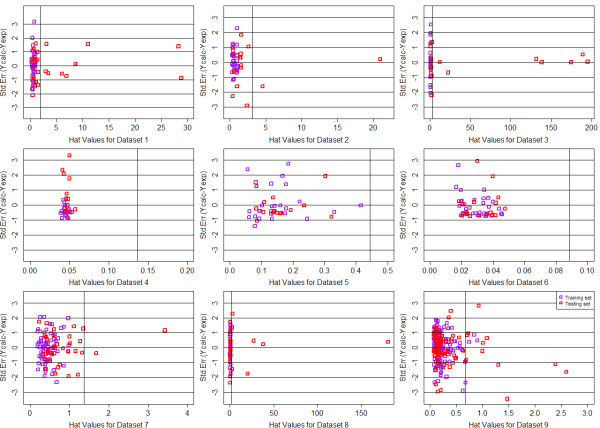
**Williams plots of 9 QSAR models based on STL with General Descriptor**.

In order to define the domain of applicability of our QSAR model, the Williams plots were drew for each QSAR model, based on MTL and STL with GD and DI feature space respectively. For each dataset, 65% of the whole data was chosen as the train data and the remains were used as testing. The plots were summarized from Figure 8, 9, 10 and 11. Each plot contains the compounds represented as the training set (purple squares) and test set (red squares). It can be clearly seen that most of the compounds fall into their corresponding application domain, which indicated that for both MTL and STL the chemical molecules are following a well-defined domain of applicability. It is worth noting that for most of those molecules following outside the DOA, their values of *Y *axis are generally fall inside the 3*σ *line, which indicated that, to a large extent, the predictions of our model for the outliers may still be reliable. In addition, very few compounds in several models indeed fall outside the 3*σ *line, however, they are close to the line thus can be retained.

After building the QSAR model, the weight of the DL features for MTL on 3 HIV datasets, 6 HCV datasets and all 9 datasets respectively were calculated, Sparse MTL in this case was trained with 50% of the data for each task and tested with the remaining data. The features ranking were showed in Figure 8, 9 and 10. It should be noted that the GD feature space will not be adopted for feature ranking due to its indirectly mapping of biological meanings.

**Figure 12:**
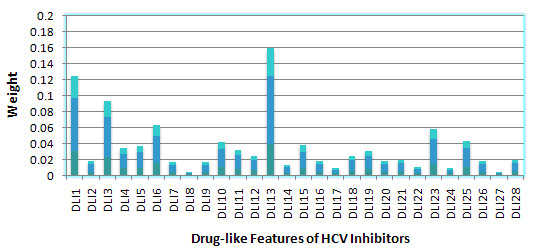
**Weights of Drug-like features of HIV inhibitors**.

**Figure 13:**
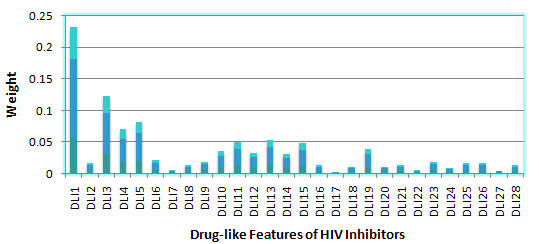
**Weights of Drug-like features of HCV inhibitors**.

**Figure 14:**
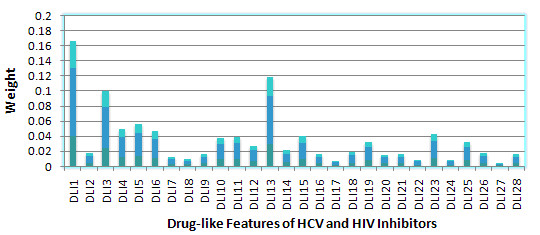
**Weights of Drug-like features of HIV-HCV inhibitors**.

Table 2 were concluded from Figure 12, 13 and 14 to provide a joint feature ranking of the DL compound descriptors. Top five features were selected to guide our structure-activity prediction as implemented with structure-activity report (*SAReport*) in MOE [27]. These features are # of non-H, # of 2-degree acyclic atoms, degree of cyclization, # of non-H polar bonds and # of rotatable bonds.

**Table 2:** A joint feature ranking of the DL compound descriptors.

Features	Ranking values
# of non-H	0.167148

# of 2-degree cyclic atoms	0.118946

degree of cyclization	0.100268

# of non-H polar bonds	0.056622

# of rotatable bonds	0.049770

# of carbons in cap fragments	0.047428

# of cap fragments	0.043447

# of 3-degree cyclic atoms	0.040664

# of N and O atoms	0.038882

# of H-bond acceptors	0.038103

# of fragments	0.033010

maximum cap fragment size	0.032459

# of 2-degree acyclic atoms	0.027549

# of 3-degree acyclic atoms	0.021673

# of 3-level bonding patterns	0.018798

total SSSR size	0.017162

total number of 3-8 membered rings	0.017162

# of cyclic fragments	0.016538

# of 1-level bonding patterns	0.016382

# of H-bond donors	0.016217

total number of 3 to 8 unsaturated rings	0.016072

# of aromatic systems	0.014930

# of N with # of H > 0	0.012320

# of hydroxyl groups	0.009301

maximum SSSR size	0.008863

# of linkers	0.008807

# of 2-level bonding patterns	0.006729

total number of 3 to 8 saturated rings	0.004750

*SAReport *was applied to present a direct instruction on how to modify the structure of a compound to make it to be a better multi-target co-inhibitor of HIV and HCV. The top structures were selected with their ranking of affinity improvements according to various modification mechanisms. As indicated from Table 3, 4, 5, 6 and 7, the modifications of compound based on these structure features are expected to be a feasible way for efficient multi-target co-inhibitor design. Generally, the following useful modification strategies were obtained, i.e., (1) Improving the # of non-H; (2) Improving the # of 2-degree acyclic atoms; (3) Improving the degree of cyclization; (4) Improving the # of non-H polar bonds and (5) Improving the # of rotatable bonds. These modifications will improve the binding affinities of HIV and HCV inhibitors respectively. Such common mechanism for improving the inhibitor's affinity on different anti-HIV or anti-HCV targets is consistent with our ranking of joint feature selection obtained by multi-target co-inhibitors QSAR modelling.

**Table 3:** More # of non-H, # of non-H polar bonds and # of rotatable bonds could increase the potency of HIV Protease Inhibitors.

Precursor		Structure	pKi
#17 pKi: 9.28			+ 2.11% (× 25.3)

#17 pKi: 9.28			+ 2.04% (× 25.3)

#17 pKi: 9.28			+ 1.95% (× 25.2)

**Table 4:** More # of 2-degree cyclic atoms, degree of cyclization and # of non-H polar bonds could increase the potency of HIV Reverse Transcriptase Inhibitors.

Precursor		Structure	pKi
#5 log(1/EC50): 8.3			+ 3.88% (× 27.2)

#5 log(1/EC50): 8.3			+ 3.77% (× 26.8)

#5 log(1/EC50): 8.3			+ 3.39% (× 26.4)

#5 log(1/EC50): 8.3			+ 3.39% (× 26.3)

#5 log(1/EC50): 8.3			+ 3.36% (× 26.3)

#5 log(1/EC50): 8.3			+ 3.31% (× 26.6)

#5 log(1/EC50): 8.3			+ 3.32% (× 26.3)

**Table 5:** More # of non-H, # of 2-degree cyclic atoms, degree of cyclization and # of non-H polar bonds could increase the potency of HIV Integrase Inhibitors.

Precursor		Structure	pKi
#11 pIC50: 5.82			+ 0.75% (× 21.2)

#189 pIC50: 5.53			+ 0.74% (× 21.2)

#188 pIC50: 4.43			+ 0.73% (× 21.3)

**Table 6:** 5 More # of non-H, # of non-H polar bonds and # of rotatable bonds could increase the potency of HCV NS5B Inhibitors.

Precursor		Structure	IC50(uM)NS5B
#15 IC50(uM)NS5B: 1.64			+ 2.51% (× 25.2)

#15 IC50(uM)NS5B: 1.64			+ 2.18% (× 24.5)

#15 IC50(uM)NS5B: 1.64			+ 1.63% (× 27.5)

#15 IC50(uM)NS5B: 1.64			+ 1.19% (× 24.1)

#15 IC50(uM)NS5B: 1.64			+ 0.98% (× 24.1)

#15 IC50(uM)NS5B: 1.64			+ 0.91% (× 24.3)

#15 IC50(uM)NS5B: 1.64			+ 0.79% (× 24.2)

**Table 7:** More # of non-H, # of 2-degree cyclic atoms, degree of cyclization and # of non-H polar bonds could increase the potency of HCV NS3 Inhibitors.

Precursor		Structure	EC50(uM)NS3
#1 EC50(uM)NS3-2: 0.35			+ 1.94% (× 21.3)

#1 EC50(uM)NS3-2: 0.35			+ 0.55% (× 17.5)

#1 EC50(uM)NS3-2: 0.35			+ 0.50% (× 17.5)

#1 EC50(uM)NS3-2: 0.35			+ 0.50% (× 17.5)

#1 EC50(uM)NS3-2: 0.35			+ 0.37% (× 17.5)

#1 EC50(uM)NS3-2: 0.35			+ 0.37% (× 17.5)

#1 EC50(uM)NS3-2: 0.35			+ 0.37% (× 17.5)

#1 EC50(uM)NS3-2: 0.35			+ 0.33% (× 17.5)

#1 EC50(uM)NS3-2: 0.35			+ 0.23% (× 17.5)

It should be noted that these features are well be commonly important for the multiple scaffolds with each inhibiting an individual target derived from our MTL model, and they can be necessarily integrated together to guide the synthesis of a single scaffold against multiple targets, which guarantee that one individual compound could hold its co-inhibitor ability for both virus targets.

## Conclusions

A Multi-target computational screening of HIV-HCV co-inhibitors with a MTL paradigm was carried out in our study. Compared to our previous work [31], it is improved mainly in two aspects: (1) It integrated both HIV and HCV data sources to enhance significantly the identification of lead inhibitors for HIV-HCV co-inhibitor drugs development. (2) A novel accelerated gradient descent algorithm based MTL model was incorporated into the multi-target QSAR modeling with more efficiency in both convergence speed and learning accuracy. In summary, the computational pipeline presented here provided an efficient way to identify and design inhibitors that simultaneously and selectively bind to multiple targets multiple viruses with high affinity.

Future researches on multi-target QSAR analysis could be done to address the compound description issue with more kinds of feature descriptors. Also the investigations on the integration and fusion mechanisms of multi-view feature spaces in compound representation could be conducted. Recently developed transfer learning technologies [36] in machine learning community may help to handle such cases efficiently. Furthermore, the underline mechanisms of HIV-HCV co-infection as well as the synthesis of the co-inhibitors based on our study are definitely worthy for long-term perusing.

## Competing interests

The authors declare that they have no competing interests.

## Authors' contributions

QL and HZ carried out the designing of the whole computational algorithm and drafted the manuscript. LL was responsible for the algorithm implementation. XC was responsible for the algorithm design. RZ and ZC conceived the study and participated in the design and coordination of the analyses. All authors read and approved the final manuscript.
